# Predictive Ability of the Estimate of Fat Mass to Detect Early-Onset Metabolic Syndrome in Prepubertal Children with Obesity

**DOI:** 10.3390/children8110966

**Published:** 2021-10-26

**Authors:** Valeria Calcaterra, Elvira Verduci, Annalisa De Silvestri, Vittoria Carlotta Magenes, Francesca Siccardo, Laura Schneider, Sara Vizzuso, Alessandra Bosetti, Gianvincenzo Zuccotti

**Affiliations:** 1 Pediatric Department, “V. Buzzi” Children’s Hospital, 20154 Milano, Italy; elvira.verduci@unimi.it (E.V.); vittoria.magenes@unimi.it (V.C.M.); francesca.siccardo@unimi.it (F.S.); laura.schneider@asst-fbf-sacco.it (L.S.); sara.vizzuso@unimi.it (S.V.); alessandra.bosetti@asst-fbf-sacco.it (A.B.); gianvincenzo.zuccotti@unimi.it (G.Z.); 2 Pediatric and Adolescent Unit. Department of Internal Medicine, University of Pavia, 27100 Pavia, Italy; 3 Department of Health Sciences, University of Milano, 20142 Milano, Italy; 4 Biometry & Clinical Epidemiology, Scientific Direction, Fondazione IRCCS Policlinico San Matteo, 27100 Pavia, Italy; a.desilvestri@smatteo.pv.it; 5 Department of Biomedical and Clinical Science “L. Sacco”, University of Milano, 20157 Milano, Italy

**Keywords:** pediatric obesity, fat mass, adiposity index, metabolic syndrome, children, adolescents, overweight

## Abstract

Body mass index (BMI), usually used as a body fatness marker, does not accurately discriminate between amounts of lean and fat mass, crucial factors in determining metabolic syndrome (MS) risk. We assessed the predictive ability of the estimate of FM (eFM) calculated using the following formula: FM = weight − exp(0.3073 × height^2^ − 10.0155 ×d-growth-standards/standards/body-mass-index-for-age-bmi-for-age weight− 1 + 0.004571 × weight − 0.9180 × ln(age) + 0.6488 × age^0.5^ + 0.04723×male + 2.8055) (exp = exponential function, score 1 if child was of black (BA), south Asian (SA), other Asian (AO), or other (other) ethnic origin and score 0 if not, ln = natural logarithmic transformation, male = 1, female = 0), to detect MS in 185 prepubertal obese children compared to other adiposity parameters. The eFM, BMI, waist circumference (WC), body shape index (ABSI), tri-ponderal mass index, and conicity index (C-Index) were calculated. Patients were classified as having MS if they met ≥ 3/5 of the following criteria: WC ≥ 95th percentile; triglycerides ≥ 95th percentile; HDL-cholesterol ≤ 5th percentile; blood pressure ≥ 95th percentile; fasting blood glucose ≥ 100 mg/dL; and/or HOMA-IR ≥ 97.5th percentile. MS occurred in 18.9% of obese subjects (*p* < 0.001), with a higher prevalence in females vs. males (*p* = 0.005). The eFM was correlated with BMI, WC, ABSI, and Con-I (*p* < 0.001). Higher eFM values were present in the MS vs. non-MS group (*p* < 0.001); the eFM was higher in patients with hypertension and insulin resistance (*p* < 0.01). The eFM shows a good predictive ability for MS. Additional to BMI, the identification of new parameters determinable with simple anthropometric measures and with a good ability for the early detection of MS, such as the eFM, may be useful in clinical practice, particularly when instrumentation to estimate the body composition is not available.

## 1. Introduction

Childhood obesity is a global health issue with a considerable growth in prevalence in the last four decades [1]. According to a World Health Organization report, in 2016, more than 340 million children and adolescents worldwide were in a condition of excess body weight. The global prevalence of overweight and obesity in males and females aged 5–19 has risen from 4% in 1975 to 18% in 2016 [1,2,3].

Pediatric obesity exposes affected patients to a higher risk of short- and long-term complications [4,5,6], including metabolic syndrome (MS). MS describes the clustering of cardio-metabolic risk factors, such as abdominal obesity, insulin resistance (IR), hypertension, and dyslipidemia, which increases the risk of developing cardiovascular diseases and type 2 diabetes mellitus in adulthood. Due to the lack of a generally accepted definition of MS in pediatric patients, a wide range—between 0.3% and 26.4%—of MS prevalence estimates has been reported [7]. De Ferranti and Cook reported a 3- to 5-fold higher MS prevalence in pubertal adolescents compared with prepubertal children [8,9] due to the relationship between MS and IR, which is strongly influenced by puberty. The degree of obesity, body composition, and body fat distribution are important factors in determining MS risk. 

Body mass index (BMI) is usually used as a marker of body fatness; moreover, it does not accurately discriminate between amounts of lean and fat mass (FM). Furthermore, height squared provides poor height standardization of weight in children with short or tall stature [10]. Additional adiposity indices, including the body shape index (ABSI), the tri-ponderal mass index (TMI), and the conicity index (Con-I), have also been considered for body composition evaluation and metabolic risk detection [11,12,13,14,15,16,17,18,19,20].

More recently, a new prediction model based on height, weight, age, sex, and ethnicity has been proposed to predict FM in children, and has obtained a high accuracy compared with BMI [21,22]. It is a simple method based on auxological parameters that does not require instruments to assess body composition [21].

Defining new parameters for the early detection of MS and therefore potential cardiovascular risk may be useful in clinical practice to better identify high-risk pediatric patients and to adopt tailored monitoring.

The aim of this study was to assess the ability of the estimate of FM (eFM) to detect early-onset MS in prepubertal children with obesity. A comparison with other adiposity parameters was also considered.

## 2. Patients and Methods

### 2.1. Patients

We retrospectively studied 185 Caucasian obese prepubertal children (110 females and 75 males) aged < 8 years with a BMI that exceeded the 97th percentile for their age and sex [23]. Patients were referred to the outpatients’ clinics of Children’s Hospital Vittore Buzzi, Milano, and San Paolo Hospital University of Milan for obesity by their general practitioner or primary care pediatrician between January 2019 and May 2021. Exclusion criteria were known secondary obesity syndromes, the use of any medications, and concomitant chronic or acute illnesses.

As a control group, 50 healthy normal-weight (BMI < 75th percentile [23]) prepubertal subjects (26 females and 24 males) aged < 8 years, who attended our clinic for auxological examination where height and weight are routinely collected, were included.

The institutional ethics committee approved the study (protocol numbers 2015/ST/135 MI, 2020/ST/234 MI) and it was conducted in accordance with the Helsinki Declaration of 1975, as revised in 2008.

### 2.2. Methods

#### 2.2.1. Clinical Evaluation

Physical examination of the patients included the evaluation of weight, height, and pubertal stage, the latter according to Marshall and Tanner [24] (prepubertal characteristics corresponding to Tanner stage 1), and the measurement of systolic (SBP) and diastolic (DBP) blood pressure. As adiposity indexes, we considered: BMI, waist circumference (WC), eFM, waist-to-height ratio (WHtR), ABSI, TMI, visceral adiposity index (VAI), and Con-I.

Weight, height, and blood pressure are recorded as previously described [25].

BMI was calculated as body weight (kilograms) divided by height (meters squared). According to BMI, the children were divided into two groups:

- Obese: BMI > 97th percentile for age and sex;
- Normal weight: <75th percentile for age and sex.

BMI was transformed into BMI z scores using WHO reference values [23].

The eFM was calculated using the following formula: FM = weight − exp(0.3073 × height^2^ −10.0155 × d-growth-standards/standards/body-mass-index-for-age-bmi-for-age weight−1 +0.004571 × weight − 0.9180 × ln(age) + 0.6488 × age^0.5^ + 0.04723 × male + 2.8055) [22] (exp = exponential function, ln = natural logarithmic transformation, score 1 if child was of black (BA), south Asian (SA), other Asian (AO), or other (other) ethnic origin and score 0 if not, ln = natural logarithmic transformation, male = 1, female = 0).

Waist circumference was measured as previously described [25]. WHtR, ABSI, TMI, VAI, and Con-I were calculated as follows:

- WHtR = WC/Ht;
- ABSI = 1000*WC*Wt ^−2/3^*Ht^5/6^ [26];
- TMI = weight (kg)/height (m)^3^ [17];
- Con-I = WC/(0.109*(Wt/Ht)^0.5^) [27];
- VAI [28].
- Boy = [WC/(39.68 + (1.88 × BMI))] × (TG/1.03) × (1.31/HDL-C); Girl = [WC/(36.58 + (1.89 × BMI))] × (TG/0.81) × (1.52/HDL-C).

#### 2.2.2. Biochemical Parameters

In all patients, blood measures included fasting blood glucose (FBG), insulin, total cholesterol, high-density lipoprotein (HDL)-cholesterol, and triglycerides (TG). Insulin resistance (IR) was calculated by homeostasis model assessment for insulin resistance (HOMA-IR) [29].

At present, there is still no international consensus for a widely accepted definition of MS in pediatrics, particularly for children under 10 years. As previously reported [30], to diagnose MS, we used the modified criteria from the National Cholesterol Education Program’s Adult Treatment Panel III (NCEP-ATPIII) [31], the World Health Organization, and the International Diabetes Federation [32], adopting the same parameters but with adapted cut-offs according to percentile for age and sex.

Patients were classified as having MS if they met ≥3 of the 5 following criteria: WC ≥ 95th percentile; TG level ≥ 95th percentile; HDL-cholesterol level ≤ 5th percentile; SBP and/or DBP ≥ 95th percentile; FBG ≥ 100 mg/dL and/or impaired insulin sensitivity (ISI) defined as HOMA-IR ≥ 97.5th percentile for age and sex [33].

We used percentile values because this scoring system is useful to stratify children at risk and it is well accepted in pediatrics [34,35,36]; at or above the 95th percentile for WC, TG, SBP, and/or DBP (and at or under 5th percentile for HD-cholesterol) there is a need for urgent intervention [34].

As a marker of gluco-metabolic derangement, a pathological level of FBG and/or IR was considered because impaired fasting glucose is rare in childhood and IR precedes glucose abnormalities, playing an important role in the transition from normal glucose tolerance to impaired glucose tolerance [37]. The euglycemic-hyperinsulinemic clamp is the gold standard for measuring IR, but this method is invasive, time-consuming, and difficult to apply to pediatric patients.

## 3. Statistical Analysis

Quantitative values were described as the mean and standard deviation (sd) or the median and interquartile range if not normally distributed (Shapiro–Wilk test). Qualitative variables were described as counts and percentages. Comparisons between groups were made with a chi-square test for qualitative variables and with a t-test or Mann–Whitney test for quantitative data. Associations between quantitative variables were assessed with the Pearson correlation coefficient (Pearson’s r). Diagnostic accuracy in predicting MS was evaluated using the area under the ROC curve (AUC) for BMI, eFM, and other adiposity indices. The AUC was compared with the methods of DeLong [38].

## 4. Results

The clinical and metabolic data of the normal-weight and obese children are reported in Table 1.

All adiposity indices were significantly higher in obese than in normal-weight children (*p* < 0.001), except for ABSI values. FBG (*p* < 0.001), insulin (*p* < 0.001), HDL-cholesterol (*p* < 0.001), TG (*p* < 0.001), and SBP (*p* = 0.02) were higher in obese compared to normal-weight children. No differences in total cholesterol and diastolic pressure values were noted.

### 4.1. Metabolic Syndrome Data

MS occurred in 18.9% (35/185) of children with obesity and in 0% of normal-weight children (*p* < 0.001), with a higher prevalence recorded in females compared to males (21/119, 60% vs. 14/75, 40%, *p* = 0.005). In Table 2, the clinical and biochemical data in males and females with or without MS are reported. Fasting blood glucose, HOMA-IR, SBP, and DBP were significantly different in non-MS and MS children in both sexes (*p* < 0.05).

As reported in Table 3, significant differences were present in BMI (*p* < 0.001), eFM (*p* < 0.001), VAI (*p* < 0.001), ABSI (*p* = 0.04), fasting blood glucose, insulin and triglycerides (*p* < 0.001), HOMA-IR (*p* < 0.001), HDL-cholesterol (*p* < 0.01), SBP (*p* = 0.002), and DBP (*p* < 0.001) values between non-MS and MS obese children.

In children with MS, we noted hypertension in 34.3% of patients, pathological values of total cholesterol and HDL-cholesterol in 40% and 22.9%, respectively, hypertriglyceridemia in 31.43%, and IR and pathological fasting blood glucose in 88.6% and 5.7%, respectively.

### 4.2. Estimate FM and Clinical and Metabolic Parameters

Significantly higher values of the eFM were found in obese compared to normal-weight children (mean values 14.8 ± 0.34 vs. 8.2 ± 0.46, *p* < 0.001).

The eFM was significantly correlated with BMI (r = 0.69, *p* < 0.001), WC (r = 0.32, *p* < 0.01), the ABSI (r = −0.47, *p* < 0.001), and the Con-I (r = −0.35, *p* < 0.001).

Higher eFM values were present in the MS compared to the non-MS group (*p* < 0.001); in particular, the eFM was higher in pediatric patients with hypertension (*p* < 0.004) and IR (*p* < 0.001); no difference in the eFM was detected for other metabolic pathological conditions.

### 4.3. Area under Curve

In Table 4, the area under the curve using ROC analysis for each adiposity parameter is reported. The eFM showed a higher predictive ability for MS compared to other indices, in particular for hypertensive component detection.

## 5. Discussion

We assessed the ability of the eFM to detect early-onset MS in prepubertal children with obesity. In our population, 18.9% of the prepubertal participants showed MS. The eFM was correlated with other adiposity indices and metabolic parameters, and a higher eFM was present in MS compared to non-MS subjects. The eFM shows a good ability for MS detection and could be proposed as a useful tool to estimate FM when it is not possible to measure body composition.

The prevalence of childhood obesity has increased dramatically since the 1980s [1,39,40]. Particularly, class I obesity has increased up to nearly 22% among prepubertal children [1,41]. Alarmingly, in 2019, 38.2 million children under the age of 5 were estimated to be overweight or obese [41]. Even though a plateau or even a decline in prevalence rates has been reported for several developed countries during the past 10 years, obesity and related complications also represent a public health problem in pediatrics [42].

Early obesity onset is associated with the early development of metabolic and cardiovascular risk factors, which, leading to atherosclerosis and vascular changes, contribute to an increased risk of premature death in adulthood [43,44]. Importantly, the earlier a metabolic alteration is developed, the worse the outcome [44,45,46,47,48,49], and pediatric onset of adulthood obesity has been shown to have higher morbidity and mortality rates compared to adult-onset obesity [49].

The prevalence of MS in prepubertal children, which is lower than the prevalence reported in older children and adulthood, has been reported as ranging from 13% to 20% [50,51,52,53]. These results are in accordance with the data we found.

Detecting MS during the prepubertal period is relevant, considering the strong correlation between the timing of obesity onset and the risk of becoming an obese adolescent and, progressively, an obese adult, along with the consequent pathological implications [54]. Geserick et al. evaluated changes in BMI over time in more than 50,000 children, from 0 to 18 years, and found out that more than 80% of children with obesity at the age of 4 became obese adolescents [55]; evaluating retrospectively obese adolescents, they found that nearly 30% of them were obese at 5 years of age [55], with the greatest increase in BMI values observed between 2 and 6 years of age [55].

We confirmed a higher prevalence of MS in prepubertal children with obesity compared to normal-weight patients, supporting the finding that obesity is already a precursor to metabolic problems in children of a very young age [54,56]. The identification of new parameters determinable with anthropometry and with a good ability for the early detection of MS may be useful in the pediatric clinical practice, where the biochemical profile is not always available. In our study, BMI, VAI, and eFM showed a satisfying ability to detect early-onset MS; however, VAI requires the lipid profile to be calculated and, therefore, compared to BMI and eFM, it has more limited use.

The eFM is a newly validated prediction model created to estimate fat mass levels in children by using simple anthropometric measures. Importantly, this model accurately discriminates between lean and fat mass [21], and it is determined by an equation considering height, weight, age, sex, and ethnicity. These are readily available markers that do not require expensive or complex forms of assessment. This method is thus more suitable for routine clinical practice than bioelectrical impedance analysis (BIA), as the instrumentation required for the latter is not always available [57,58,59].

The eFM has been recently associated with increased intima media thickness and hypertrophy of the carotid wall, demonstrating the significant and independent role played by fat mass in the development of vascular abnormalities and highlighting the importance of treating this condition to preserve vascular health [22]. A high percentage of FM in obese children has also been associated with elevated BP levels [60,61], in particular systolic BP [61], especially in girls and older-aged (16–17 years old) children [61,62], and with cardiometabolic risk factors, such as IR, LDL, TG, and total cholesterol levels [63].

In our work, we found a significantly higher eFM in obese children compared to normal-weight subjects; moreover, the eFM was significantly correlated with other indices of obesity, such as BMI, WC, the ABSI, and the Con-I.

We found a positive correlation between the eFM and MS. Specifically, the eFM was significantly higher in patients with IR and hypertension, which are important features of MS and cardiovascular risk factors [64]. Even though the causative role of insulin in the development of hypertension is still questioned, the association between IR and hypertension is well documented in pediatrics [65], and IR is considered the primary pathogenic mechanism underlying MS and a link between obesity and disease [66,67]. The relationship between the eFM and IR suggests the potential role of these parameters in the early detection of metabolic derangement.

The strict correlations we found between the eFM and the metabolic parameters cited above are relevant, as obesity and its consequences are some of the most serious pediatric health problems [68]. Furthermore, the eFM calculated using our model is more suitable for routine use compared to BIA, and is a more accurate measure of FM compared to BMI [22]; the latter, although widely used, does not discriminate between lean and fat mass in children [69], and has been considered a worse predictor of cardiovascular risk factors with respect to FM in adults [70]. The eFM could be an important tool for the surveillance and prevention of obesity-related complications.

We recognize that there are some limitations to this study, starting with its retrospective nature. Secondly, a relatively small sample size was used; a larger cohort of children could therefore be useful to confirm these results. Additionally, we considered only Caucasian children; however, determining the predictive value of the eFM in a multi-ethnic population could be useful to support its strength; further validation in different populations is thus needed. Finally, a comparison between the eFM and FM estimated from bioimpedance with regard to their predictive ability to detect MS could improve the validity of the findings.

## 6. Conclusions

MS is already associated with obesity in very young children, and an early MS detection may decrease the risk of cardiovascular diseases and type 2 diabetes mellitus in adulthood. Additional to BMI, the identification of new parameters determinable with simple anthropometric measures and with a good ability for the early detection of MS, such as the eFM, may be useful in clinical practice, particularly when the instrumentation to estimate body composition is not available.

## Figures and Tables

**Table 1:** Clinical and biochemical data in obese and normal-weight children.

Parameters	All	Normal Weight (*n* = 50)	Obesity (*n* = 185)	*p* *
Age (years)	7.01 ± 0.09	7.06 ± 0.18	6.73 ± 0.10	0.08
Body mass index (kg/m^2^)	22.76 ± 0.26	16.95 ± 0.30	24.32 ± 0.21	<0.001
Body mass index z-score	2.08 ± 0.14	−0.40 ± 0.30	3.10 ± 0.09	<0.001
Waist circumference (cm)	72.71 ± 0.65	60.56 ± 1.13	75.98 ± 0.56	<0.001
Waist circumference/height ratio	0.57 ± 0.006	0.45 ± 0.007	0.60 ± 0.005	<0.001
Estimate fat mass (%)	13.40 ± 0.33	8.22 ± 0.46	14.79 ± 0.34	<0.001
Visceral adiposity index	2.26 ± 0.10	1.57 ± 0.16	2.44 ± 0.11	<0.001
Body shape index	0.08 ± 0.005	0.07 ± 0.007	0.08 ± 0.006	0.15
Tri-ponderal mass index	18.08 ± 0.24	12.72 ± 0.20	19.52 ± 0.19	<0.001
Conicity index	1.24 ± 0.008	1.16 ± 0.012	1.26 ± 0.008	<0.001
Homeostasis model assessment for insulin resistance	1.77 ± 0.10	0.92 ± 0.23	1.99 ± 0.10	<0.001
Fasting blood glucose (mg/dL)	77.38 ± 0.65	71.88 ± 1.43	78.86 ± 0.70	<0.001
Insulin (UI/mL)	9.02 ± 0.44	5.35 ± 0.84	9.98 ± 0.49	<0.001
Total cholesterol (mg/dL)	155.09 ± 1.78	154.80 ± 2.04	156.26 ± 3.60	0.74
HDL-cholesterol (mg/dL)	49.06 ± 0.80	48.03 ± 0.93	53.15 ± 1.32	<0.001
Triglycerides (mg/dL)	68.32 ± 2.17	51.62 ± 3.53	72.91 ± 2.48	<0.001
Systolic blood pressure (mmHg)	102.30 ± 0.76	98.87 ± 1.31	103.2 ± 0.88	0.02
Diastolic blood pressure (mmHg)	61.37 ± 0.55	59.36 ± 1.08	61.90 ± 0.63	0.06

* *p*-values: normal weight vs. obesity. Data are reported as mean ± standard deviation.

**Table 2:** Clinical and biochemical data in males and females with or without metabolic syndrome (MS).

Parameters	Males (*n* = 75)		Females (*n* = 110)	
	Non-MS (*n* = 61)	MS (*n* = 14)	Non-MS (*n* = 89)	MS (*n* = 21)
Age (years)	6.91 ± 0.12	7.14 ± 0.35	6.49 ± 0.12	6.91 ± 0.23
Body mass index (kg/m^2^)	24.0 ± 0.33	26.54 ± 0.67 *	24.0 ± 0.32	25.29 ± 0.53
Body mass index z-score	3.34 ± 0.20	2.64 ± 0.18	3.03 ± 0.14	3.2 ± 0.26
Waist circumference (cm)	76.98 ± 1.11	75.90 ± 1.69	74.70 ± 0.74	77.10 ± 1.73
Waist circumference/height ratio	0.61 ± 0.01	0.58 ± 0.01	0.61 ± 0.01	0.62 ± 0.01
Estimate of fat mass (%)	14.16 ± 0.47	18.13 ± 0.86 *	14.28 ± 0.54 *	16.71 ± 1.08
Visceral adiposity index	1.57 ± 0.11	2.39 ± 0.28 *	2.76 ± 0.18	3.68 ± 0.32
Body shape index	0.082 ± 0.001	0.074 ± 0.002 *	0.081 ± 0.008	0.081 ± 0.017
Tri-ponderal mass index	18.99 ± 0.30	20.30 ± 0.85	19.61 ± 0.30	20.24 ± 0.51
Conicity index	1.28 ± 0.01	1.18 ± 0.03 *	1.26 ± 0.01	1.27 ± 0.02
Homeostasis model assessment for insulin resistance	1.40 ± 0.09	3.08 ± 0.47 *	1.91 ± 0.16	3.39 ± 0.25 *
Fasting blood glucose (mg/dL)	77.70 ± 1.26	86.21 ± 1.97 *	77.29 ± 0.93	84.90 ± 1.74 *
Insulin (UI/mL)	7.15 ± 0.43	14.25 ± 1.89	9.86 ± 0.80	16.08 ± 1.05
Total cholesterol (mg/dL)	156.46 ± 3.33	169.42 ± 6.81	151.50 ± 2.97	154.52 ± 7.08
HDL-cholesterol (mg/dL)	49.58 ± 1.43	42.57 ± 2.48 *	48.98 ± 1.45	43.85 ± 3.03
Triglycerides (mg/dL)	63.09 ± 3.87	89.92 ± 8.51 *	72.96 ± 3.48	90.57 ± 8.80 *
Systolic blood pressure (mmHg)	102.37 ± 1.39	112.57 ± 2.68 *	100.23 ± 1.19	111.85 ± 2.96 *
Diastolic blood pressure (mmHg)	60.37 ± 0.90	69.00 ± 3.04 *	60.30 ± 0.82	68.23 ± 2.22 *

Data are reported as mean ± standard deviation. * *p* < 0.05 non-MS vs. MS.

**Table 3:** Clinical and biochemical data in patients with and without metabolic syndrome.

Parameters	Non-MS (*n* = 150)	MS (*n* = 35)	*p*
Age (years)	6.66 ± 0.09	7.0 ± 0.19	0.10
Body mass index (kg/m^2^)	24.0 ± 0.23	25.79 ± 0.42	<0.001
Body mass index z-score	3.13 ± 0.11	3.0 ± 0.18	0.57
Waist circumference (cm)	75.60 ± 0.63	77.26 ± 1.23	0.25
Waist circumference/height ratio	0.60 ± 0.10	0.57 ± 0.01	0.80
Estimate of fat mass (%)	14.23 ± 0.37	17.28 ± 0.73	<0.001
Visceral adiposity index	2.26 ± 0.12	3.16 ± 0.24	<0.001
Body shape index	0.081 ± 0.006	0.079 ± 0.014	0.04
Tri-ponderal mass index	19.36 ± 0.22	20.26 ± 0.45	0.07
Conicity index	1.27 ± 0.009	1.23 ± 0.02	0.13
Homeostasis model assessment for insulin resistance	1.70 ± 0.10	3.27 ± 0.24	<0.001
Fasting blood glucose (mg/dL)	77.46 ± 0.75	85.42 ± 1.29	<0.001
Insulin (UI/mL)	8.75 ± 0.51	15.35 ± 0.98	<0.001
Total cholesterol (mg/dL)	153.53 ± 2.22	160.48 ± 5.13	0.18
HDL-cholesterol (mg/dL)	49.23 ± 1.03	43.34 ± 2.05	0.01
Triglycerides (mg/dL)	68.84 ± 2.61	90.31 ± 6.20	<0.001
Systolic blood pressure (mmHg)	101.10 ± 0.91	112.14 ± 2.05	<0.001
Diastolic blood pressure (mmHg)	60.33 ± 0.60	68.54 ± 1.78	<0.001

MS: metabolic syndrome. Data are reported as mean ± standard deviation.

**Table 4:** Area under curve using ROC analysis for all adiposity parameters.

Parameters	ROC Area (95% Confidence Interval)			
	Metabolic Syndrome	Blood Pressure	Hyperglycemia or IR	Dyslipidemia
Estimate of fat mass	0.71 (0.62–0.80)	0.69 (0.59–0.80)	0.70 (0.61–0.77)	0.59 (0.46–0.66)
Body mass index (kg/m^2^)	0.70 (0.61–0.80)	0.65 (0.54–0.76)	0.71 (0.63–0.78)	0.60 (0.51–0.69)
Waist circumference (cm)	0.58 (0.47–0.68)	0.65 (0.54–0.76)	0.61 (0.52–0.70)	0.50 (0.41–0.59)
Waist circumference/height ratio	0.49 (0.37–0.60)	0.42 (0.29–0.56)	0.54 (0.44–0.63)	0.49 (0.40–0.58)
Visceral adiposity index	0.70 (0.62–0.79)	0.45 (0.33–0.57)	0.70 (0.64–0.80)	0.72 (0.65–0.80)
Body shape index	0.38 (0.27–0.49)	0.38 (0.25–0.51)	0.43 (0.33–0.52)	0.40 (0.39–0.51)
Tri-ponderal mass index	0.62 (0.52–0.72)	0.57 (0.46–0.67)	0.63 (0.54–0.71)	0.59 (0.50–0.68)
Conicity index	0.42 (0.30–0.53)	0.41 (0.27–0.54)	0.47 (0.37–0.56)	0.42 (0.32–0.51)

## Data Availability

The data presented in this study are available on request from the corresponding author.
